# Oncogenes overexpressed in metastatic oral cancers from patients with pain: potential pain mediators released in exosomes

**DOI:** 10.1038/s41598-020-71298-y

**Published:** 2020-09-07

**Authors:** Aditi Bhattacharya, Malvin N. Janal, Ratna Veeramachaneni, Igor Dolgalev, Zinaida Dubeykovskaya, Nguyen Huu Tu, Hyesung Kim, Susanna Zhang, Angie K. Wu, Mari Hagiwara, A. Ross Kerr, Mark D. DeLacure, Brian L. Schmidt, Donna G. Albertson

**Affiliations:** 1 Bluestone Center for Clinical Research, New York University College of Dentistry, 421 First Avenue, Room 233W, New York, NY 10010 USA; 2 Department of Oral and Maxillofacial Surgery, New York University College of Dentistry, New York, NY 10010 USA; 3 Department of Epidemiology and Health Promotion, New York University College of Dentistry, New York, NY 10010 USA; 4 Applied Bioinformatics Laboratories, New York University Langone Medical Center, New York, NY 10016 USA; 5 New York University College of Dentistry, New York, NY 10010 USA; 6 Department of Radiology, New York University School of Medicine, New York, NY 10016 USA; 7 Department of Oral and Maxillofacial Pathology, Radiology and Medicine, New York University, New York, NY 10010 USA; 8 Division of Head and Neck Surgery and Oncology, New York University School of Medicine, New York, NY 10016 USA

**Keywords:** Oral cancer, Metastasis

## Abstract

Oral cancer patients experience pain at the site of the primary cancer. Patients with metastatic oral cancers report greater pain. Lack of pain identifies patients at low risk of metastasis with sensitivity = 0.94 and negative predictive value = 0.89. In the same cohort, sensitivity and negative predictive value of depth of invasion, currently the best predictor, were 0.95 and 0.92, respectively. Cancer pain is attributed to cancer-derived mediators that sensitize neurons and is associated with increased neuronal density. We hypothesized that pain mediators would be overexpressed in metastatic cancers from patients reporting high pain. We identified 40 genes overexpressed in metastatic cancers from patients reporting high pain (n = 5) compared to N0 cancers (n = 10) and normal tissue (n = 5). The genes are enriched for functions in extracellular matrix organization and angiogenesis. They have oncogenic and neuronal functions and are reported in exosomes. Hierarchical clustering according to expression of neurotrophic and axon guidance genes also separated cancers according to pain and nodal status. Depletion of exosomes from cancer cell line supernatant reduced nociceptive behavior in a paw withdrawal assay, supporting a role for exosomes in cancer pain. The identified genes and exosomes are potential therapeutic targets for stopping cancer and attenuating pain.

## Introduction

Each year 22,000 new cases of oral cancer occur in the United States with tongue being the most common site (40–50% of cases). While the incidence of oral cancer has been decreasing due to reduction in smoking, the incidence of tongue cancer has increased in young (< 50 years) men and women^1^. Metastasis is the primary determinant for survival. Clinically node negative (cN0) patients are managed either by observation or by prophylactic removal of the cervical lymph nodes (elective neck dissection) at the time of surgical resection of the cancer. Failure to surgically resect occult neck metastasis results in significantly lower 5-year survival^2^. Cervical lymph node involvement in oral cancer patients is currently assessed with physical examination and radiographic methods including CT, MRI and PET. Meta-analyses evaluating these imaging modalities conclude that the methods may miss > 20% of occult metastases^2^. Elective neck dissection is the prefered treatment. Most patients, however, do not benefit from surgical removal of lymph nodes. For example, the incidence of occult metastasis for early stage tongue cancer ranges from 8 to 46%^3^. Thus, there is a need for improved preoperative assessment of metastatic risk.

Oral cancer patients suffer severe chronic and mechanically-induced pain at the site of the cancer^4–6^. Opioids are initially effective, but dose escalation is required and side effects reduce quality of life. Most patients no longer experience pain after surgical removal of the cancer^7^. Oral cancer pain is attributed to sensitization or activation of primary afferent neurons by mediators released from the cancer and microenvironment. Cancers also induce sprouting of sensory and sympathetic nerves into the microenvironment^6,8^. We reported previously that patients with metastatic (N+) cancers experienced greater pain^9^.

Here we evaluated association of oral cancer patient reported pain, metastasis, and molecular differences between N+ cancers from patients experiencing high levels of pain and N0 cancers. We find that preoperative pain signals N+ cancers. Proteins encoded by genes differentially expressed in N+ cancers from patients with high levels of pain are enriched for functions in extracellular matrix organization and angiogenesis. They are reported in exosomes. Depletion of extracellular vesicles from cancer cell line conditioned media reversed conditioned media-induced thermal and mechanical allodynia in a mouse model. We suggest that the genes carried by exosomes have potential to interact with the cancer microenvironment to induce pain by activating sensory neurons.

## Results

### Patients with oral cavity cancer experience significant function-related pain prior to surgical resection of the cancer

**Figure 1:**
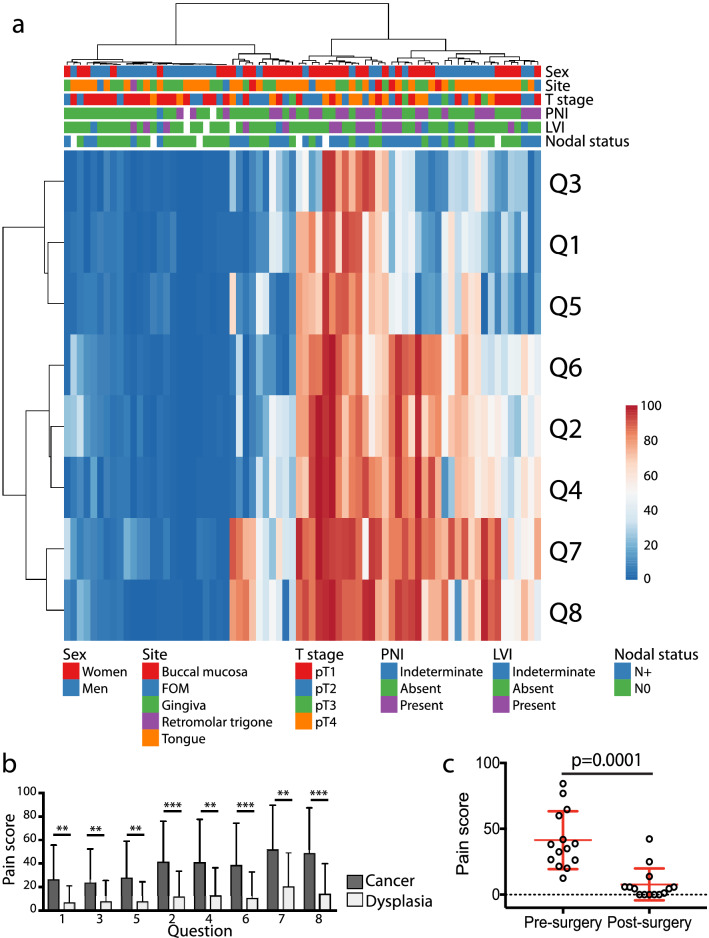
Oral cancer patients provide varied responses to the UCSFOCPQ. (**a**) Clustering (Euclidian distance with Ward linkage) of patients based on pain questionnaire responses q1–q8. Responses to individual questions are represented in rows and patients in columns. Patient characteristics are indicated above the heatmap. Patients clustered into four groups—patients reporting low pain scores in response to all questions, high pain on questions 7 and 8, high pain on questions 2, 4, 6, 7 and 8 or high pain in response to all questions. (**b**) Cancer patients experience greater pain than dysplasia patients. Shown are responses (mean and SD) to questions on the UCSFOCPQ from cancer and dysplasia patients. **p < 0.01, ***p < 0.001, Kruskal–Wallis *H* test with Dunn's multiple comparisons test. (**c**) Validation of the UCSFOCPQ for oral cancer patients as measured with the average of the eight questions. Shown are pain scores from individual patients with the mean and standard deviation indicated by the red bars. Following surgery patients experienced less pain (Mann–Whitney *U* test). Data from reference^7^.

We used the University of California Oral Cancer Pain Questionnaire (UCSFOCPQ) to evaluate preoperative pain experienced by 72 oral cancer patients (Supplementary Table S1). The UCSFOCPQ is the only validated instrument specifically tailored to oral cancer pain^7,9^. Responses from oral cancer patients clustered into four groups according to intensity of pain reported on the UCSFOCPQ (Fig. 1a). Lowest mean scores were reported on questions 1, 3 and 5 (spontaneous sensation) and higher scores on questions 2, 4 and 6 (function related pain), consistent with the previous observation that oral cancer patients suffer function related mechanical pain^9^. Highest mean pain scores were reported on questions 7 and 8 (Fig. 1b). By contrast, patients with precancers, oral epithelial dysplasia (n = 28, Supplementary Table S2), rarely reported pain and scores on the UCSFOCPQ were significantly lower for all questions compared to cancer patients (Fig. 1b), consistent with our previous report^10^.

Although the eight questions address different content areas, we determined that the responses to these items did not vary independently in the cohort of 72 patients. The median Pearson correlations between each pair of questions was 0.75 with a range from 0.62 to 0.95. A principal components analysis showed a single eigenvalue greater than 1 (6.44) that accounted for 80.5% of variability in these responses. Considered as a single scale, Cronbach’s alpha was 0.96, which was unchanged by omitting any single question. Therefore, we used the average of the eight questions, as the measure of the response to the UCSFOCPQ. The single score also discriminated among patients tested in the previously published validation cohort^7^ (Fig. 1c).

### Node positive patients report higher pain scores

**Figure 2:**
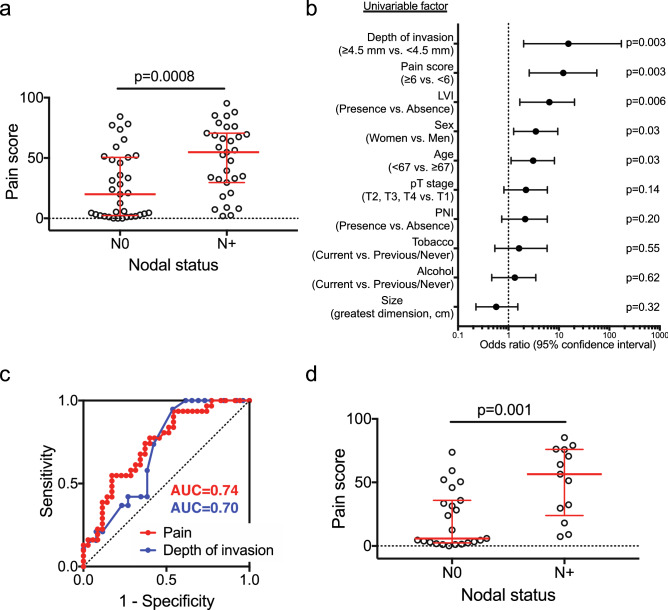
Pain and depth of invasion predict metastasis in oral cancer patients. (**a**) Patient reported pain is associated with metastasis (Mann–Whitney *U* test). Bars represent median and interquartile range. (**b**) Univariate analysis of metastasis predictors. Bars = 95% confidence intervals. (**c**) Receiver operating characteristic curve analysis was performed with pain score (n = 66 patients) and depth of invasion (n = 45 patients). (**d**) Pain reported by cT1N0 and cT2N0 patients (n = 36) is associated with metastasis (Mann–Whitney *U* test). Bars represent median and interquartile range.

Nodal status at one-year follow-up was available for 66 patients (N+  = 31 and N0 = 35). Higher pain scores were reported by patients with N+ than N0 cancers (*p* = 0.0008, Fig. 2a). Depth of invasion measured on the resection specimen is considered the best predictor of metastasis in oral cancer patients^11^. In addition, presence of perineural invasion (PNI) and lymphovascular invasion (LVI have been reported to predict metastasis^12,13^. Information on depth of invasion, PNI and LVI was available for 45, 63 and 57, respectively, of the 66 patients whose nodal status was confirmed after at least one year of follow up (Supplementary Table S1). We found significant associations between metastasis and depth of invasion (*p* = 0.02) and LVI (*p* = 0.006), but not PNI. Age (*p* = 0.03) and sex (*p* = 0.03) were also associated with metastasis, but not tumor size (greatest dimension), T stage (T1 vs. T2, T3, T4), use of alcohol or tobacco (Fig. 2b). Receiver operating characteristic curve analysis (Fig. 2c) showed that the area under the curve (AUC) was similar for pain score and depth of invasion (0.74 and 0.70, respectively). Using the Youden Index to determine optimal cutoffs, pain score (< 6) and depth of invasion (< 4.5 mm) each predicted low metastatic risk with high sensitivity and high negative predictive values, both better than PNI and LVI (Table 1). By multivariate analysis, we found that depth of invasion and pain do not independently report nodal status [OR = 1.04, (CI 0.991–1.098), *p* = 0.10 and OR = 1.02, (CI 0.991–1.055), *p* = 0.18, respectively], suggesting that patients’ responses on the UCSFOCPQ provide a preoperative indicator of depth of invasion.

**Table 1:** Test characteristics of pain and pathological features for prediction of metastasis.

	Pain score (< 6)	Depth of invasion (< 4.5 mm)	LVI	PNI
Sensitivity	0.94	0.95	0.48	0.52
Specificity	0.46	0.46	0.88	0.67
Positive predictive value	0.60	0.56	0.75	0.58
Negative predictive value	0.89	0.92	0.68	0.61

### Pain scores identify clinically node negative (cN0) patients at low risk of metastasis prior to surgery

Standard of care assessment of oral cancer patients uses preoperative imaging (MRI, CT, PET) and clinical exam to stage patients as cN+ or cN0. Due to the limited sensitivity of current imaging modalities, however, an elective neck dissection is recommended for early stage cT1N0 and cT2N0 patients^2^. Since pathologic features cannot be accurately assessed prior to surgery, we asked whether pain scores of cT1N0 and cT2N0 patients (n = 36) predicted final nodal status as determined by pathology (pN0 or pN+) or for patients who did not receive a neck dissection (pNx), they remained free of metastasis after one year. Clinically node negative T1 and T2 patients with metastasis reported greater pain (Fig. 2d). In building the model, we also considered information known prior to surgery, including sex, age, alcohol and tobacco history and race/ethnicity. Nodal status was independently associated with pain scores and age (*p* = 0.02 and *p* = 0.03, respectively). Using the cutoff for low pain score determined above (< 6), 12 (33.3%) of the 36 cT1N0 and cT2N0 patients were correctly assigned to be N0. Six (50%) of these patients had received a neck dissection. In addition to imaging and physical exam, a decision tree analysis recommends a neck dissection if there is > 20% probability of occult metastasis^14^. The analysis takes into consideration probability of occult metastasis, therapeutic efficacy, quality-of-life and patient preferences. In the future, pain score might be added to a decision tree analysis for the management of the cT1T2N0 neck.

### Gene expression profiling identified 40 genes implicated in mediating oral cancer pain and metastasis

**Figure 3:**
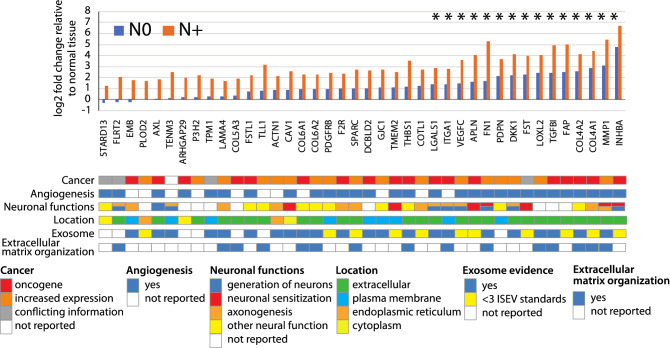
The pain and metastasis genes encode extracellular proteins enriched for functions in extracellular matrix organization, angiogenesis, oncogenesis and axonogenesis. Shown are expression levels of the pain and metastasis genes relative to normal tissue in pN+ cancers (n = 7) and other cancers (n = 12) determined to be N0 by pathological evaluation of the neck dissection specimen (pN0) or by lack of nodal disease after one year for patients who did not have a neck dissection (i.e., those staged pNx). Associations of the genes with cancer, angiogenesis, neuronal functions (generation of neurons (GO: 0048699), neuronal sensitization, axonogenesis or other neural functions), cellular location, whether genes have been reported in exosomes and association with extracellular matrix organization are indicated below the plot. Presence in exosomes in human tissues or cells is shown according to reported number ($\ge$ 3 or < 3) of five International Society for Extracellular Vesicles (ISEV) minimal experimental requirements for definition of extracellular vesicles. Some of the pain and metastasis genes (by definition more highly expressed in N+ cancers than N0 cancers and normal tissue) are also overexpressed in N0 cancers compared to normal tissue (indicated by asterisks). Genes are shown in ascending order of expression in N0 cancers.

To investigate the association of metastasis and pain, we used transcriptome profiling to identify candidate cancer-derived pain mediators. We examined gene expression in an RNA-seq dataset comprised of N+ (n = 7), N0 (n = 12) and normal (n = 5) oral tissues from tongue and gingiva. Pain scores were available for 17 of the 19 cancers (N+  = 7, N0 = 10). We hypothesized that N+ cancers from patients reporting high pain scores would overexpress pain mediators. We compared expression in five N+ cases with mean pain scores > 60 to ten N0 cancers from patients who had completed the UCSFOCPQ (N+ vs. N0, Supplementary Fig. S1). This analysis found 427 genes that were differentially expressed (FDR < 0.05, Supplementary Table S3). Next, we identified genes from this list that were also differentially expressed relative to normal tissue (n = 264 genes—120 increased, 144 decreased abundance). We considered genes with mean expression of at least 30 counts and log2 fold change $\ge$ 1.3 to be candidate pain and metastasis inducing genes (n = 79). We further filtered this list by manual inspection to eliminate 39 genes with high mean level of expression due to gene amplification, for example, to yield a set of 40 pain and metastasis genes (Fig. 3 and Supplementary Table S4). A similar analysis of the 144 down regulated genes identified 52 down regulated genes (Supplementary Table S4). We noted that for the majority of the pain and metastasis genes, abundance also increased in N0 cancers compared to normal tissue (Fig. 3). The increase was significant for 18/40 genes. For each cancer, we tallied the number of pain and metastasis genes that were overexpressed compared to normal tissue (log2 fold change ≥ 1.3). Numbers ranged from 6 to 40 of the pain and metastasis genes.

### Candidate cancer-derived pain mediators are enriched for protein–protein interactions and functions in extracellular matrix organization and vasculogenesis/angiogenesis

Functional enrichments (Gene Ontology, GO categories) and protein–protein interactions (PPI) were identified using the STRING^15^ database (Supplementary Fig. S1 and Supplementary Table S5). This analysis revealed a PPI enrichment value of *p* < 1.0e−16. The top GO biological process was extracellular matrix organization (15 genes, FDR = 3.99e−14). The top 10 GO processes were enriched for vasculogenesis/angiogenesis (in total, 18 different genes), including circulatory system development (n = 17 genes, FDR = 4.7e−11), blood vessel morphogenesis (n = 13 genes, FDR = 1.72e−10), vascular development (n = 14 genes, FDR = 1.72e−10), angiogenesis (n = 12 genes, FDR = 1.72e−10), tube development (n = 16 genes, FDR = 2.97e−10) and tube morphogenesis (n = 14 genes, FDR = 1.86e−09). Further manual curation using PubMed searches identified 11 additional genes involved in vasculogenesis and/or angiogenesis, including, for example, *LAMA4* (increased expression in tumor vessels in renal and colon cancer^16^), *LGALS1* (sprouting angiogenesis^17^), and *F2R* (PAR1) and *MMP1* (MMP1/PAR1 pro-angiogenic signaling^18^)—total 72.5% (29/40) of genes (Fig. 3, Supplementary Table S6). Thirty-nine genes have been reported to be oncogenes or overexpressed in cancer, with 23 reported in head and neck (HNSCC, n = 5) or oral cancer (n = 18). A similar analysis of 52 less abundant genes in N+ cancers from patients with pain yielded no significant PPIs or GO biological process enrichments (Supplementary Fig. S1).

### Pain and metastasis genes have potential to modulate nociception and neuronal sprouting

Functional enrichment analysis of the pain and metastasis genes identified nine genes associated with generation of neurons (GO: 0048699, FDR = 0.016) (Fig. 3, Supplementary Table S5). We queried PubMed for potential neuronal functions of the pain and metastasis genes. Associations were found for 30 pain and metastasis genes. Seven genes were reported in association with pain or neuronal sensitization. For example, four genes (*FN1*, *INHBA*, *MMP1* and *TMEM2*) increase excitability of the transient receptor potential cation channel subfamily V member 1 (TRPV1)^19–22^. Trigeminal nociceptive neurons expressing TRPV1 are implicated in oral cancer pain^23^. Fourteen genes are associated with sprouting of neurites (axonogenesis, neuritogenesis) and include, for example, organization of an extracellular matrix that favors neuronal outgrowth (*e.g*., *LAMA4*)^24,25^ or neuronal guidance (e.g., *FLRT2*)^24^. Ten genes have other neuronal functions (Fig. 3, Supplementary Table S6).

### Expression of genes encoding neurotrophic factors and axon guidance molecules differs among N+ cancers, N0 cancers and normal tissues

We further queried the RNA-seq data for expression of cancer-derived mediators with potential to induce neuronal sprouting. We examined expression of secreted neurotrophic factors (neurotrophins and glial cell line derived neurotrophic family (GDNF) genes) and axon guidance molecules (semaphorins, ephrins, ephrin receptors, netrins, slits, teneurins, fibronectin leucine rich transmembrane (FLRT) family and repulsive guidance molecules) (Supplementary Table S7). Forty-three genes from these families of neurotrophic and axon guidance genes were expressed in the RNA-seq dataset (normalized counts > 30). Two of these 43 guidance genes (*FLRT2*, *TENM3*) were included in the pain and metastasis gene set. Hierarchical clustering with these 43 neurotrophic factors and axon guidance genes separated normal tissues, N+ and N0 cancers (Supplementary Fig. S2). The number of differentially expressed neurotrophic and axon guidance genes (log2 fold change ≥ |1.3| relative to normal tissue) in N+ cancers was greater than in N0 cancers (N+ mean = 12.3, N0 mean = 8, *p* = 0.001 t-test). We find that pain score correlated with the number of differentially expressed neurotrophic and axon guidance genes in a cancer (Spearman’s r = 0.53, *p* = 0.028).

### Pain and metastasis genes are extracellular and reported in exosomes

Using the STRING^15^, g:Profiler^26^, SecretomeP 2.0a^27^, DeepLoc^28^, COMPARTMENTS^29^ and ExoCarta^30^ databases, 90% of the genes (36/40) were predicted to be located in the extracellular region, plasma membrane (e.g., Gene Ontology categories GO:0005576, cellular component, extracellular region, GO:0005886: plasma membrane), secreted according to the signal peptide or SecretomeP scores or present in exosomes (Fig. 3 and Supplementary Tables S5 and Supplementary S8).

### Oral cancer cell lines release exosomes with pain and metastasis genes as cargo

**Figure 4:**
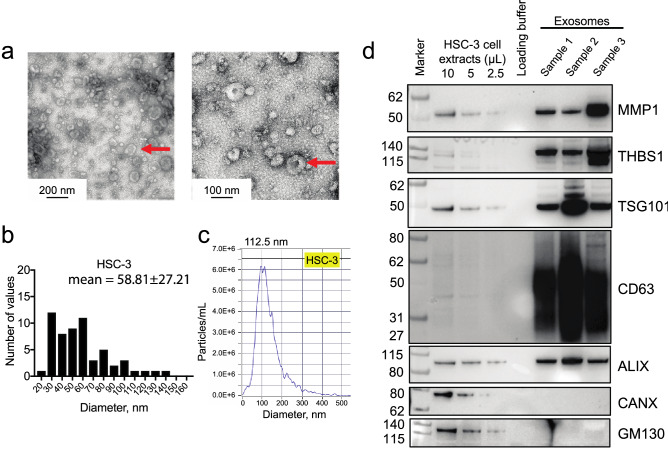
Extracellular vesicles released by HSC-3 cells have characteristics of exosomes and carry pain and metastasis genes as cargo. (**a**) Representative transmission electron micrographs of EVs isolated from conditioned media of HSC-3. Images were taken at 45,000× (left) and 92,00× (right) magnification. Isolated vesicles display cup-shaped morphology and membrane layer characteristic of exosomes (arrows). (**b**) The diameters of vesicles from HSC-3 were measured on micrographs taken at 92,000× (4–6 fields). (**c**) Representative nanoparticle tracking analysis (NTA) of the distribution of measured EV hydrodynamic diameter (mode indicated above the trace). Extracellular vesicle sizes as measured from electron micrographs and by NTA are typical of exosomes. (**d**) Isolated EVs express exosome endocytic marker proteins TSG101 and ALIX, and tetraspanin, CD63. Calnexin (CANX, endoplasmic reticulum marker) and GM130 (cis-Golgi network marker) were not detected. Exosome samples were obtained from cells grown under normoxic conditions. Pain and metastasis gene proteins, MMP1 and THBS1 were detected in the HSC-3 EVs.

To determine whether oral cancer cells released exosomes with pain and metastasis genes, we purified extracellular vesicles (EVs) from cell culture media from two oral tongue cancer cell lines, HSC-3 and OSC-20 by ultracentrifugation. Extracellular vesicles released by HSC-3 and OSC-20 cells have characteristics of exosomes as determined by transmission electron microscopy, nanoparticle tracking analysis and expression of exosome markers TSG101, CD63 and ALIX (Fig. 4 and Supplementary Fig. S3). By contrast, calnexin (CANX, endoplasmic reticulum marker) and GM130 (cis-Golgi network marker) were not detected. Pain and metastasis gene proteins, MMP1 and THBS1 were detected in EVs isolated from HSC-3 conditioned media. We detected expression of THBS1 in EVs from conditioned media from OSC-20 cells grown under normoxic conditions, but not MMP1, even though MMP1 was detected in OSC-20 cell lysates. Since hypoxic cells release greater numbers of exosomes and hypoxic exosome cargo (nucleic acids, proteins and lipids) differs from that of normoxic cells^31^, we asked whether MMP1 was expressed in EVs from OSC-20 cells grown under hypoxic conditions. We detected MMP1 in hypoxic OSC-20 EVs and increased expression of THBS1.

### Depletion of oral cancer cell line EVs reduces nociceptive behavior in a preclinical cancer model

**Figure 5:**
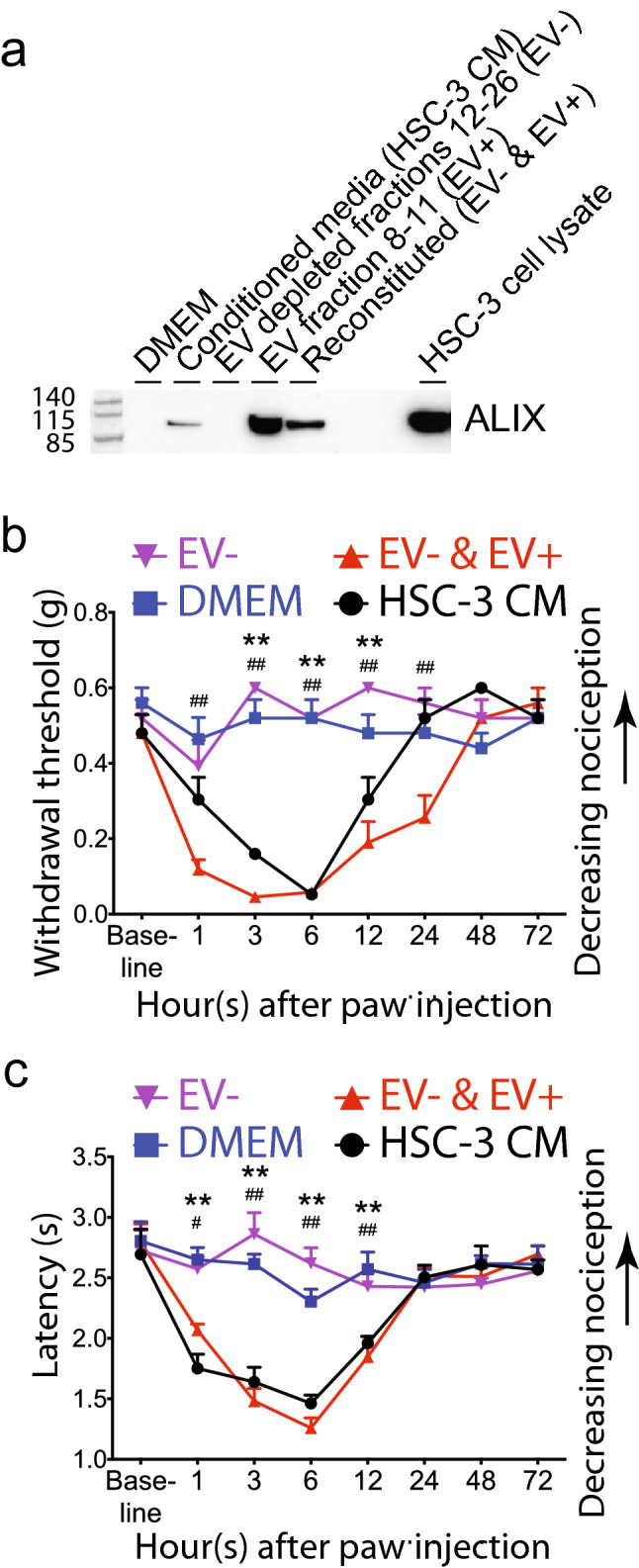
Depletion of exosomes from oral cancer cell line supernatant reduces mechanical allodynia and abolishes thermal hyperalgesia. (**a**) Western blot showing expression of EV maker, ALIX in conditioned media from tongue cancer cell line HSC-3 grown under normoxia (HSC-3 CM), media alone (DMEM), DMEM depleted of EVs by SEC (EV−) or reconstituted with EVs (EV− and EV+). To obtain sufficient material for western blots, 8 mL of conditioned media were processed by eight SEC runs. Fractions 8–11 were individually combined and concentrated to 50 µL. The allodynic effect of samples injected into the right hind paw was measured by assaying induced mechanical nociception (**b**) and thermal hyperalgesia (**c**) at baseline (before injection) and at the indicated times after injection. *p < 0.05; **p < 0.01 DMEM versus HSC-3 CM; ^##^*p* < 0.05 EV− versus EV− & EV+. Two-way ANOVA with Tukey’s multiple comparisons test. The experiment was performed twice with independent groups of animals.

To test whether oral cancer cell line EVs induce cancer pain, we isolated EVs by size exclusion chromatography (Supplementary Fig. S4) and performed a mouse paw withdrawal assay. Intraplantar injection of HSC-3 oral tongue cancer cell line conditioned media is routinely used as a model to study oral cancer pain^32,33^. The cell line supernatant induces mechanical allodynia and thermal hyperalgesia as demonstrated here in Fig. 5. By contrast, when HSC-3 conditioned media depleted of EVs by size exclusion chromatography was injected into the paw, nociceptive behavioral responses to mechanical or thermal stimuli did not differ from DMEM alone (Fig. 5). Nociceptive responses to these stimuli were restored by reconstitution of the EV depleted media with the EV fraction. These data demonstrate that pain mediators are released in the EV fraction and suggest that pain mediators released in exosomes contribute to induction of oral cancer pain.

## Discussion

The pain and metastasis genes, genes overexpressed in cancers from node positive patients with pain, are enriched for functions in extracellular matrix organization and angiogenesis and reported in exosomes. The genes are among those more highly expressed in the basal and mesenchymal subtypes of HNSCC^34^. The set of pain and metastasis genes overlaps with the meta-signature of the partial epithelial-to-mesenchymal transition (p-EMT) program, a recently described independent predictor of lymph node metastasis in HNSCC^35,36^. The p-EMT program, which may be a transient state, is characterized by increased expression of extracellular matrix genes, reduced expression of epithelial genes and lack of expression of classical EMT transcription factors. The subset of cells expressing the p-EMT program are reported to localize to the edges of the cancer. Interaction of pain and metastasis genes with the tumor microenvironment is likely to be facilitated both by the external location of cells expressing the genes and by the genes being present in exosomes.

Exosomes are 30–150 nm extracellular vesicles. They are formed within the endosomal system as intralumenal vesicles of multivesicular bodies (MVBs) and are released into the extracellular space when the MVBs fuse with the plasma membrane. Exosomes have been implicated in cancer progression, including cancer cell-cancer cell communication and cancer-microenvironment interactions (recruitment and modification of fibroblasts, angiogenesis, metastasis and immunosuppression)^37^. Under hypoxic conditions, cancer cells release greater numbers of extracellular vesicles^38,39^. Vesicle cargo reflects the hypoxic state and can confer malignant properties on recipient (normoxic) cells^39^. We observed increased expression of pain and metastasis genes *MMP1* and *THBS1* in hypoxic exosomes from OSC-20. Hypoxic exosomes have been implicated in promotion of angiogenesis and metastasis^31^. Transfer of hypoxic exosomes from oral cancer cells induced normoxic cells to adopt a prometastatic phenotype^39^. Exosomes from HNSCC also promote angiogenesis^40^, neuronal sprouting^41^ and neuronal plasticity^42^.

Here, we added promotion of oral cancer pain to the roles attributed to exosomes. Depletion of EVs from cancer cell line conditioned media reversed the conditioned media induced mechanical and thermal allodynia in a mouse model. Nucleic acids, proteins and lipids with potential to induce neuronal plasticity are carried by EVs. We considered whether other, soluble mediators might be present in the EV depleted fractions, but not detected by our behavior assays. Endothelin-1 (EDN1), a secreted 21 amino acid peptide might be such a soluble mediator. EDN1 has not been reported in exosomes in the Exocarta database^30^, but was reported in EVs isolated from rat plasma^43^. EDN1 in the cancer microenvironment induces mechanical allodynia associated with growth of HSC-3 cells in the mouse hind paw^44^. EDN1 is reported in HSC-3 conditioned media at a concentration of 34 pg/mL^44^. In the experiments reported here, HSC-3 conditioned media (20 µL) injected into the paw would contain 2.7 × 10^–4^ pmol of EDN1, well below the tested 1–30 pmol concentration range reported to dose dependently induce mechanical and thermal hyperalgesia in rodents^45–47^. It appears, therefore, that soluble mediators, if present, in the EV depleted fraction are at low levels and/or their effects are below the sensitivity of the behavior assays. The composition and roles of oral cancer extracellular vesicles in relation to oral cancer pain and metastasis warrant further investigation.

Overexpressed pain and metastasis genes and differentially expressed neurotrophic and axon guidance genes may directly sensitize primary afferent sensory neurons^19–22^ and/or promote increased density and type of neuronal infiltration into the microenvironment. Sensory innervation is reported to be favored over sympathetic innervation in HNSCC^41^, pancreatic cancer^48^ and painful non-malignant conditions^49^. Increased density of innervation is associated with pain, lymph node metastasis and poor prognosis in a number of cancer types^6,8^. Nine pain and metastasis genes have roles in axonogenesis and angiogenesis. Perivascular nerve growth and angiogenesis are observed in rodent cancer models^50,51^ and correlate with disease progression and pain in a pancreatic cancer model^50^. Parallel growth of blood vessels and nerves is also a characteristic of chronic non-malignant pain conditions^52,53^.

The pain and metastasis genes may play multiple roles in cancer promotion. Matrix metalloproteinase 1 (*MMP1*), for example, may contribute to remodeling the extracellular matrix (collagenolytic activity) and activate PAR1 on cancer cells and endothelial cells to promote oncogenic signaling and angiogenesis, respectively^18^. MMP1 may contribute to cancer pain signaling by promoting neuronal sprouting^54^, activation of PAR1 to sensitize TRPV1 on nociceptors^20^ and/or by activation of protease activated receptor 2 (PAR2, *F2RL1*)^55,56^, a mediator of cancer pain signaling^6^. A functional *MMP1* polymorphism is associated with patient reported pain from lumbar disk herniation^57^ and positive nodal status in breast cancer patients^18^. Further studies will be required to understand the role of *MMP1*, PAR1 and PAR2 in oral cancer and oral cancer pain.

This first study of gene expression and oral cancer pain involved a small number of patients. Moreover, all N+ cancers were from women, allowing for a possible sex bias in our study. Further studies are needed to address these limitations. We also acknowledge that we did not demonstrate the in vivo impact of the genes on pain and metastasis or confirm the overexpression of the 40 pain and metastasis genes at the protein level. Evidence supporting putative pain mediator and oncogene roles for the genes can be found in the literature. For example, injection of INHBA into the paw elicits nociceptive behavior and release of the neuropeptide CGRP (calcitonin gene-related peptide, CALCA) from nociceptors^22,58^. Application of MMP1 to DRG neurons induces release of substance P from DRG neurons^20^. Overexpression of 19 of the genes in HNSCC has been reported as assessed by immunohistochemistry and 16 genes have been reported in association with metastasis in HNSCC or oral cancer (Supplementary Table S6). Our analysis is further supported, by reports identifying overexpression and oncogenic functions for 39 of the pain and metastasis genes in cancers, including in HNSCC and oral cancer (Supplementary Table S6). This observation is consistent with the expectation that oral cancer pain mediators released from the cancer would be putative oncogenes, not tumor suppressors.

In conclusion, we confirmed that pain is correlated with metastasis in oral cancer, and we demonstrated that pain score has potential to signal presence of metastasis prior to surgery. Pending future validation, preoperative reports of pain intensity appear to be promising indicators of risk for metastasis. Our transcriptome analysis highlighted the composition of the N+ oral cancer microenvironment for promotion of angiogenesis and axonogenesis. We suggest that the collection of pain and metastasis genes and neurotrophic and axon guidance genes differentially expressed in each cancer determines density of innervation and neuronal sensitization leading to the pain experienced by a patient. Preclinical studies implicated cancer-derived exosomes in induction of pain. The autocrine and paracrine actions of identified pain and metastasis genes (e.g., *MMP1* and PAR1), as well as communication via exosomes, are potential therapeutic targets for stopping cancer and alleviating pain.

## Methods

### Patient populations

We evaluated pain in a cohort of 72 oral cancer patients and a cohort of 28 oral dysplasia patients enrolled at the New York University Oral Cancer Center between 2010 and 2017 (Supplementary Tables S1 and S2). The study was carried out in accordance with the Code of Federal Regulations on the Protection of Human Subjects (45 CFR Part 46), the National Institutes of Health requirements for human subjects research and institutional research policies and procedures of the Institutional Review Board at New York University. All personnel involved in the conduct of this study completed Human Subjects Protection Training.

All subjects gave written informed consent. Cancer patients were included if they were receiving curative treatment for an oral cavity cancer, they had not received chemotherapy or radiation treatment for a prior cancer and they had completed the University of California Oral Cancer Pain Questionnaire (UCSFOCPQ) within 30 days prior to surgery. Dysplasia patients were included if they had completed the UCSFOCPQ within two months of biopsy or resection of the precancer. We collected demographic information, which included: age, sex, ethnicity (white, Hispanic, Africa-American, Asian), location of the oral cancer (tongue, buccal mucosa, gingiva, floor of mouth, retromolar trigone), tumor size and evidence of metastasis (based on clinical examination and radiographic imaging). From pathology reports, we obtained information on lymph node metastasis (patients who received a neck dissection), TNM stage, differentiation, tumor thickness/depth of invasion and presence of perineural invasion, lymphovascular invasion and extracapsular spread. Nodal status at 1-year follow-up was available for 66 patients–29 patients were determined to have positive nodes by examination of the neck dissection specimen immediately following surgery (pN+), whereas 37 were node negative (pN0). Neck dissection was not performed on eight patients, so that the status of the neck could not be determined (pNx). During the 1-year follow-up period, two pN0 patients developed neck disease. None of the pNx patients developed neck disease and were classified as N0, giving a final nodal status of N+  = 31 and N0 = 35.

### Pain questionnaire

The UCSFOCPQ asks patients to rate the intensity of their pain on a 0–100 point visual analogue scale (VAS) in response to eight questions. The questions differentiate spontaneous and function-related pain and determine the quality of pain. Questions 1, 3 and 5 ask about spontaneous intensity, sharpness and aching, while questions 2, 4, and 6 ask about these sensations when talking, eating or drinking. Question 7 asks about sensitivity to touch and question 8, how significantly pain interferes with function. The UCSFOCPQ was administered to cancer patients at a preoperative clinic visit before being prescribed analgesics for their oral cancer pain and before any treatment, as described previously^7^. If patients were currently taking pain medication, they were asked to refrain for 24 h. The study was approved by the New York University School of Medicine Institutional Review Board and was carried out in accordance with the NYU School of Medicine Policies and Procedures for Human Subjects Research Protection. All patients consented to participate in the study.

### RNA-seq and analysis of differentially expressed genes

Total RNA was extracted from 30 mg of fresh frozen oral cancer tissue and clinically normal contralateral gingiva or tongue tissues from oral cancer patients using the Qiagen RNeasy kit (Catalog No: 74104) following the manufacturer’s protocol including the recommended DNase treatment. Homogenized tissue lysates were prepared by using a small hand-held pestle followed by passing the lysate at least five times through a blunt 20-gauge needle fitted to an RNase-free syringe. The RNA concentration and integrity were determined using an Agilent 2100 Bioanalyzer, equipped with an RNA 6000 Nano kit (part number 5067-1511), following the manufacturer’s instructions. Ribodepleted strand specific RNA libraries were prepared from 24 total RNA samples. Samples were pooled and 125 base paired end sequencing was performed on the Illumina 2500. Sequencing results were demultiplexed and converted to FASTQ format using Illumina bcl2fastq software. The sequencing reads were adapter and quality trimmed with Trimmomatic^59^ and then aligned to the human genome (build hg38/GRCh38) using the splice-aware STAR aligner^60^. The featureCounts program^61^ was utilized to generate counts for each gene based on how many aligned reads overlap its exons. These counts were then normalized and used to test for differential expression using negative binomial generalized linear models implemented by the DESeq2 R package^62^.

### Cell lines and supernatant collection

Human oral tongue cancer cell lines, HSC-3 (Cat# JCRB0623, RRID:CVCL_1288, passages 4-12) and OSC-20 (Cat# JCRB0197, RRID:CVCL_3087, passages 4-12) were obtained from the Japanese Collection of Research Bioresources Cell Bank. The cell lines were cultured in Dulbecco’s modified Eagle’s medium (DMEM, Gibco, Waltham, MA, USA, HSC-3) or DMEM/F12 (OSC-20) supplemented with fetal bovine serum (FBS) and penicillin/streptomycin (50 U/mL) for 48 h. Cell lines were cultured in 75 cm^2^ cell culture flasks at 37 °C with 5% CO_2_, 21% O_2_ (normoxia) or in 1% O_2_ (hypoxia). When cells reached 70–80% confluency, the culture medium was replaced with media supplemented with 10% exosome-depleted fetal bovine serum (Gibco) and cultured for a further 48 h for preparation of exosomes. Cell culture supernatant was collected and used for isolation of exosomes.

### Extracellular vesicle (EV) isolation by ultracentrifugation

Cell culture supernatant was centrifuged at 300×*g* for 10 min to pellet cells, then transferred to a fresh tube and centrifuged at 2000×*g* for 20 min at 4 °C to remove dead cells. The supernatant was centrifuged at 10,000×*g* for 30 min at 4 °C to remove cell debris. Extracellular vesicles were isolated by ultracentrifugation at 120,000×*g* for 70 min at 4 °C. The pellet was washed with DPBS followed by repeat ultracentrifugation under the same conditions. The EV pellet was suspended in 40–50 µL DPBS and used immediately.

### Transmission electron microscopy

A 5 μL suspension of EVs in 2% formaldehyde in PBS was added onto a carbon coated 400 mesh Cu/Rh grid (Ted Pella Inc., Redding, CA) and stained with 1% uranyl acetate (Polysciences, Inc, Warrington, PA) in ddH_2_O. Stained grids were examined with a Thermo Fisher Talos L120C transmission electron microscope and photographed with a Gatan OneView digital camera.

### Particle size analysis

Extracellular vesicle preparations from HSC-3 and OSC-20 cell line supernatants were analyzed for size by nanoparticle tracking analysis with a Zetaview analyzer (Particle Metrix GmbH, Meerbusch, Germany) following the manufacturer’s recommendations.

### Western blotting analysis

Protein samples were separated by 4–12% Bis–Tris Gels (Invitrogen) and transferred to a 0.2 µm pore-size nitrocellulose membrane (Bio-Rad). The primary antibodies used were: mouse monoclonal anti-human MMP1 (clone MAB901, 1:1000, R&D Systems, RRID:AB_2144271), goat polyclonal anti-human THBS1 (AF3074, 1:1000, R&D Systems, RRID:AB_2201958), rabbit polyclonal anti-TSG101 (A303-507A, 1:1,000, Bethyl Laboratories Inc., RRID:AB_10971167), rabbit polyclonal anti-CD63 (PA5-78995, 1:1000, Invitrogen, RRID:AB_2746111), mouse monoclonal anti-human ALIX (clone OTI1A4, VMA00273, 1:2000, Bio-Rad PrecisionAb western blot validated), sheep polyclonal anti-GM130/GOLGA2 (AF8199-SP, 1:1000, R&D Systems), rabbit anti-calnexin (C4731, 1:2000, Sigma-Aldrich, RRID:AB_476845). Pre-stained protein ladder was from Abcam (ab234592).

### Cancer cell line conditioned media pain model

For preparation of conditioned media, HSC-3 cells were cultured in two 10 cm cell culture dishes in 10 mL of media and 10% exosome depleted FBS for 48 h (70–80% confluent). The cells were washed three times with PBS and cultured for a further 48 h in 3 mL serum-free DMEM without phenol red. Cell culture supernatant was collected, centrifuged at 300×*g* for 10 min to pellet cells. The supernatant was centrifuged at 2000×*g* for 20 min at 4 °C, 10,000×*g* for 30 min at 4 °C, and filtered through a 0.22 µm Millipore filter to remove dead cells, debris and large vesicles. One mL of media was loaded on a Sepharose CL-2B column (1.5 cm × 6.2 cm) and 0.5 mL fractions were collected. Extracellular vesicles elute in fractions 8–11 (Supplementary Fig. S3). To prepare exosome depleted and reconstituted supernatant, fractions 8–11 (EV fraction) and 12–26 (EV depleted fraction) were each concentrated to 1 mL using Vivaspin 3 kDa MWCO centrifigual concentrators. The reconstituted supernatant [Exo (+) and Exo (−)] was prepared by mixing 0.5 mL each of the EV and EV depleted fractions and concentrating the mixture to a final volume of 1 mL using Vivaspin 3 kDa MWCO centrifugual concentrators.

Following recording baseline behavior, male mice (C57BL/6 J, 8 weeks old, JAX # 000664) were arbitrarily selected to receive one of four treatments by an investigator blinded to the identity of the treatment. The mice received an intraplantar injection (20 µL) into the right hind paw under 1% isoflurane anesthesia of the following: HSC-3 supernatant (HSC-3 sup.), cell line media only (DMEM), conditioned media depleted of exosomes by size exclusion chromatography [Exo (−)], or reconstituted supernatant [Exo (−) and Exo (+)]. Based on our experience with the paw withdrawal assay, group sizes of five mice were used. Animal experiments were carried out in accordance with the recommendations of the National Institute of Health guidelines and the PHS Policy on the Humane Care and Use of Laboratory Animals. The protocol was approved by the New York University Institutional Animal Care and Use Committee.

#### Behavioral testing

Mechanical nociception was measured with calibrated von Frey filaments (Stoelting, Wood Dale, IL, USA). The mice were placed individually in a transparent chamber on a raised platform with a metal mesh floor for one hour before testing. The right hind paw was stimulated with the von Frey filament through the mesh floor of each chamber. Withdrawal threshold was defined as the gram-force sufficient to elicit a distinct paw withdrawal upon application of the von Frey filament tip. Withdrawal threshold was determined as the mean of three trials for each animal. The mechanical nociception assay was conducted before (Baseline), 1, 3, 6, 12, 24, 48, and 72 h(s) after the right plantar injection.

Thermal hyperalgesia was measured with a paw thermal stimulator (IITC Life Sciences, Woodland Hills, CA). The mice were placed in a transparent chamber on a pre-heated (29 °C) glass surface for one hour before the thermal hyperalgesia assay. A radiant heat source was focused on the right hind paw. Paw withdrawal latency was measured as the mean of three trials taken at least 5 min apart. The cut-off latency was established at 20 s to avoid tissue damage. The thermal hyperalgesia assay was conducted before (Baseline), 1, 3, 6, 12, 24, 48, and 72 h(s) after the right plantar injection. The investigators performing the mechanical nociception and thermal hyperalgesia assays were blinded to the treatment.

### Statistical analysis

We used GraphPad Prism 7 for Mac OS X (version 7.0c) for receiver operating characteristic curve analysis, to test for mean differences between groups with the Mann–Whitney *U* test and to test for differences in proportions with the Fisher Exact test. Hierarchical clustering was performed using ClustVis^63^. Logistic regression analysis was used to evaluate the simultaneous effects of pain and depth of invasion on nodal status and was computed using IBM SPSS (v24, IBM Corp., Armonk, NY, USA).

## Supplementary information

Supplementary Table S1 & S2

Supplementary Table S3

Supplementary Table S4

Supplementary Table S5

Supplementary Table S6

Supplementary Table S7

Supplementary Table S8

Supplementary Figures

## Data Availability

Data are available through NCBI Gene Expression Omnibus (GSE156178).
