# Supplementary material for: Oncogenes overexpressed in metastatic oral cancers from patients with pain: potential pain mediators released in exosomes
Source: Sci Rep. 2020 Sep 7;10:14724. doi: 10.1038/s41598-020-71298-y (PMC7477576; doi:10.1038/s41598-020-71298-y)

# Oncogenes overexpressed in metastatic oral cancers from patients with pain: potential pain mediators released in exosomes

Aditi Bhattacharya, Malvin N. Janal, Ratna Veeramachaneni, Igor Dolgalev, Zinaida Dubeykovskaya, Nguyen Huu Tu, Hyesung Kim, Susanna Zhang, Angie K. Wu, Mari Hagiwara, A. Ross Kerr, Mark D. DeLacure, Brian L. Schmidt and Donna G. Albertson

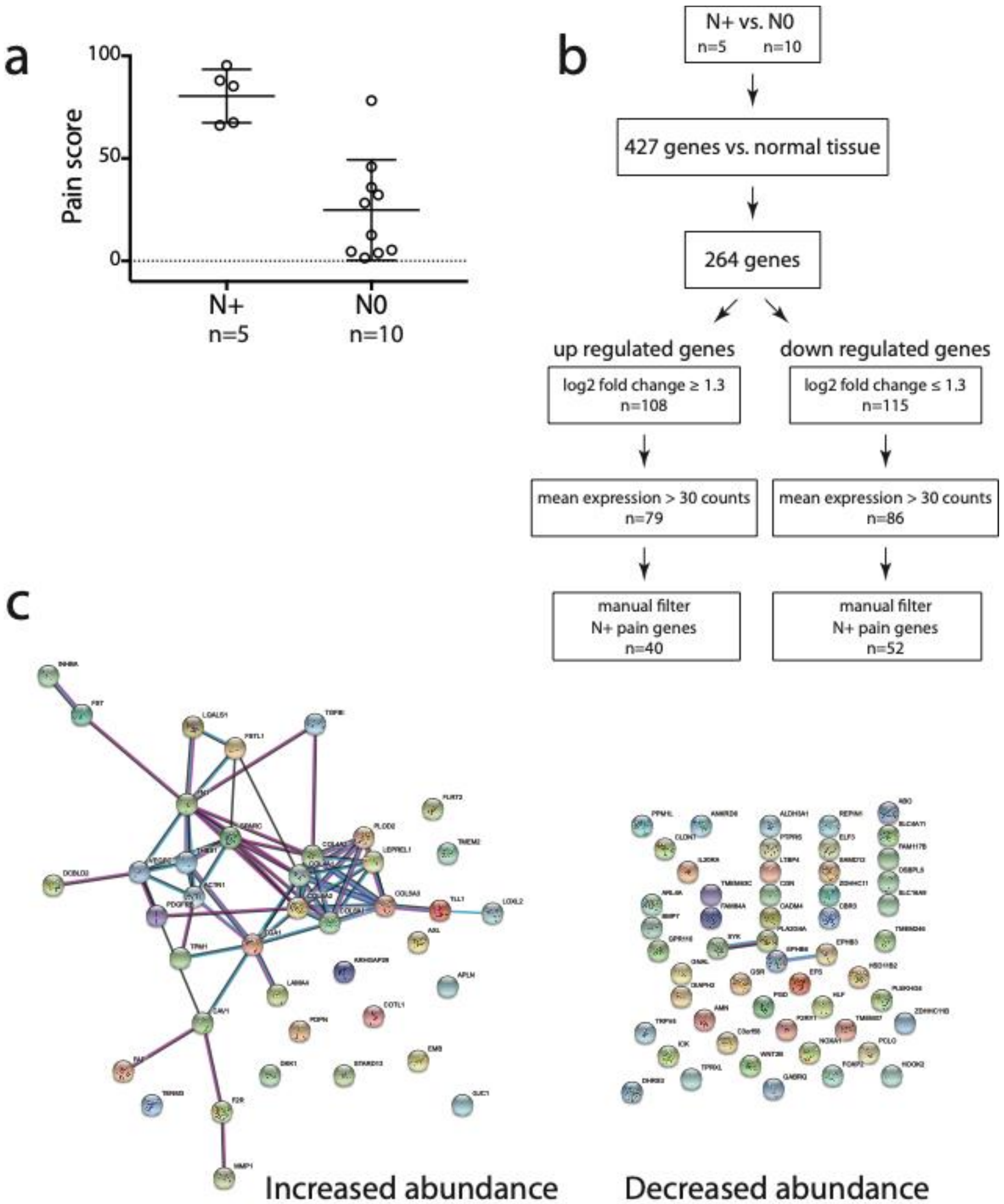


**Supplementary Figure S1**. Identification of candidate pain inducing genes in N+ cancer. Transcriptome analysis by RNA-seq was performed on 19 cancers and five normal samples. Five patients with N+ cancers and high pain scores were compared to 10 patients with N0 cancers. (**a**) Pain scores for the N+ patients with high pain (n=5) and N0 patients (n=10) included in the analysis. (**b**) Flow diagram for identification of genes with increased/decreased transcript abundance in N+ cancers compared to N0 cancers and normal tissue samples. (**c**) Networks of N+ pain genes generated by the String database for the 40 genes with increased or 52 genes with decreased abundance in N+ cancers from patients with high pain levels. Nodes are proteins. Colored nodes are first shell of interactions. White nodes are second shell of interactions. Edges represent protein-protein interactions, colored according to known interactions (from curated databases, experimental data), predicted interactions (gene neighborhoods, fusions, co-occurrence) and other information (text mining, co-expression, protein homology).

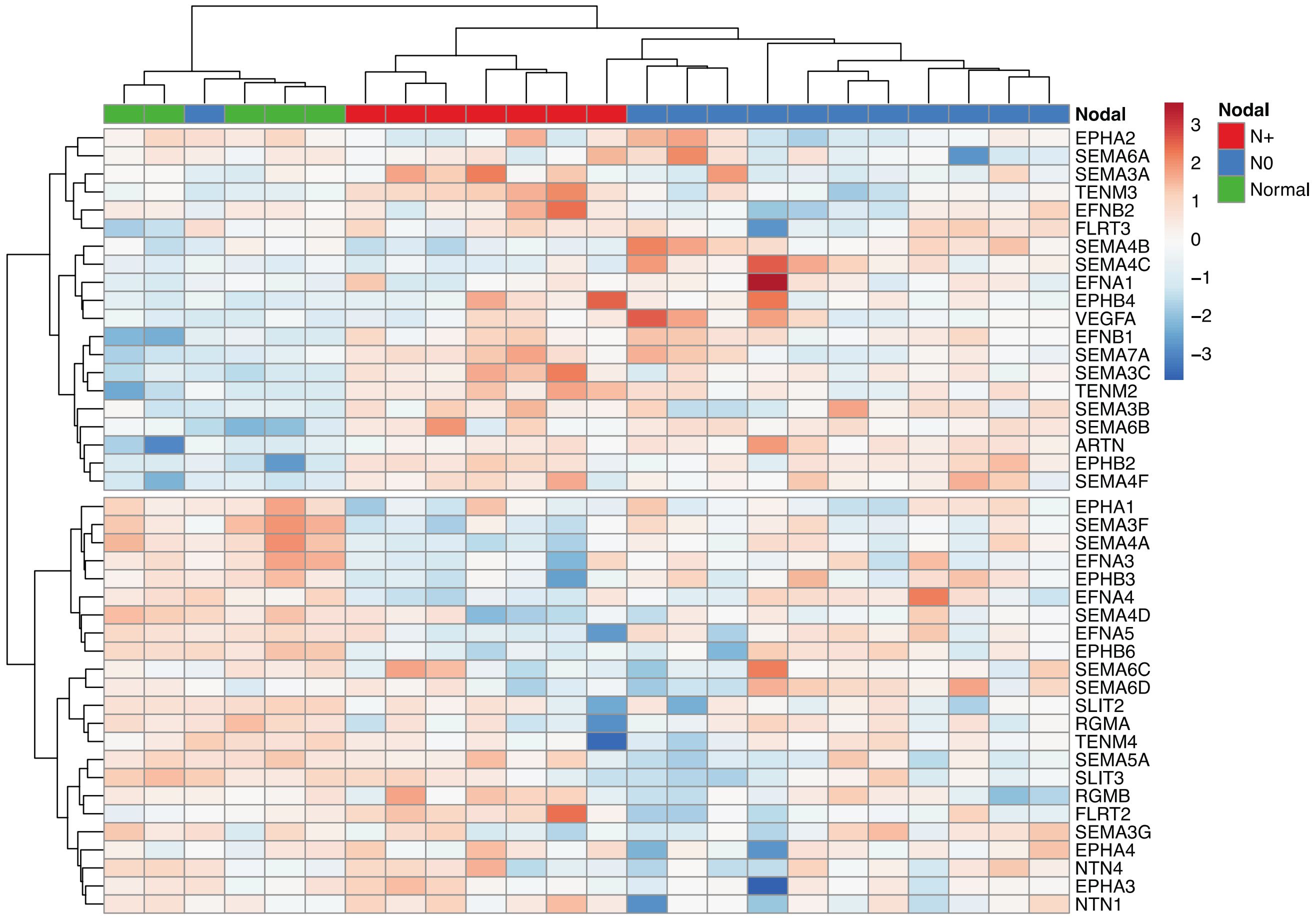


**Supplementary Figure S2**. Separation of cases and normal tissue samples by expression of neurotrophic and axon guidance genes. Clustering (Euclidian distance with Ward linkage) of patients based on log2 transformed expression of neurotrophic genes and axon guidance genes. Normal tissues and nodal status of the cancers are indicated above the heatmap.

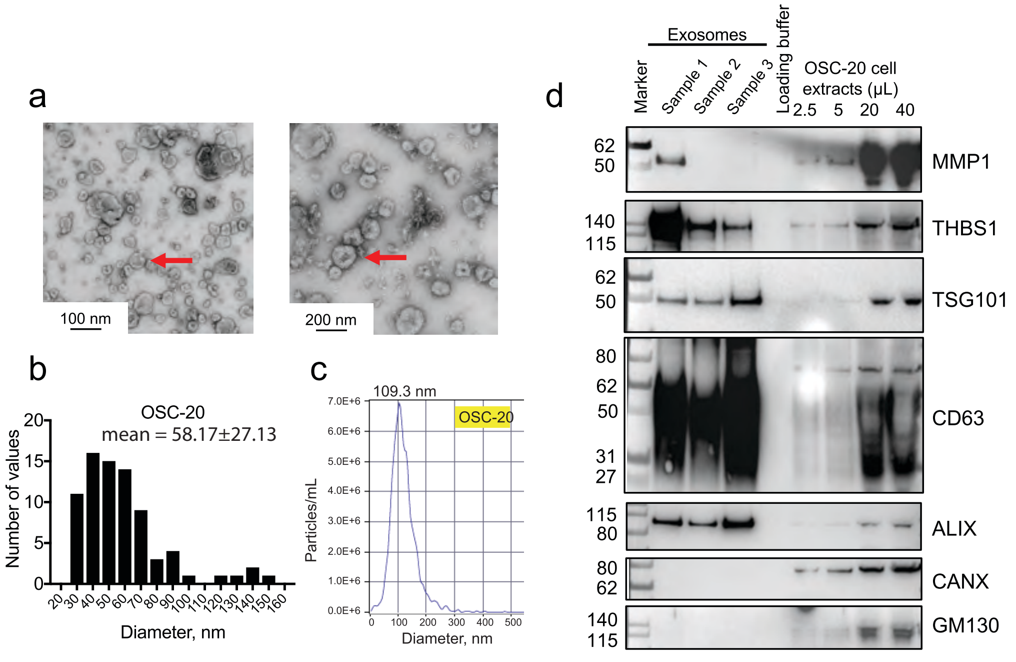


**Supplementary Figure S3**. Extracellular vesicles released by OSC-20 cells have characteristics of exosomes and carry pain and metastasis genes as cargo. (**a**) Representative transmission electron micrographs of EVs isolated from conditioned media of OSC-20. Images were taken at 45,000x (left) and 92,000x (right) magnification. Isolated vesicles display cup-shaped morphology and membrane layer characteristic of exosomes (arrows). (**b**) The diameters of vesicles from OSC-20 were measured on micrographs taken at 92,000x (4-6 fields). (**c**) Representative nanoparticle tracking analysis (NTA) of the distribution of measured EV hydrodynamic diameter (mode indicated above the trace). Extracellular vesicle sizes as measured from electron micrographs and by NTA are typical of exosomes. (**d**) Isolated EVs express exosome endocytic marker proteins TSG101 and ALIX, and tetraspanin, CD63. Calnexin (CANX, endoplasmic reticulum marker) and GM130 (cis-Golgi network marker) were not detected. Exosome sample 1 was from cells grown under hypoxic conditions, samples 2 and 3 under normoxia. Pain and metastasis gene protein, MMP1 was detected only in exosomes from hypoxic cells. THBS1 was detected in exosomes from OSC-20 conditioned media grown under normoxic and/or hypoxic conditions.

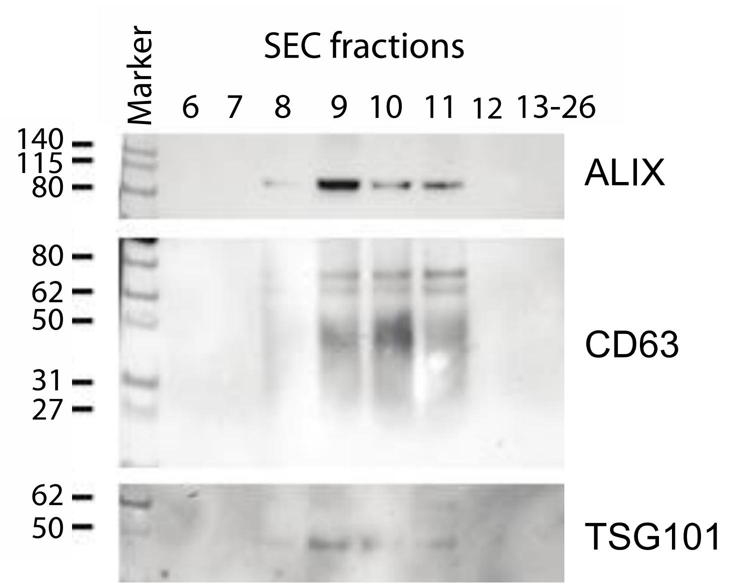


**Supplementary Figure S4.** Isolation of EVs by size exclusion chromatography. Six mL of conditioned media were fractionated by loading 1 mL aliquots on a Sepharose CL-2B column. Fractions (0.5 mL) were collected. Fractions 6, 7, 8, 9, 10, 11 and 12 were individually combined, and concentrated to 50 µL using Vivaspin 3 kDa MWCO centrifigual concentrators. Fractions 13-26 were collected together and concentrated to 50 µL for analysis. Exosome markers are detected in fractions 8-11.

# Western blots for Figures 4d and 5 and Supplementary Figures S3 and S4

Figure 4d Exosomes from HSC-3 cells

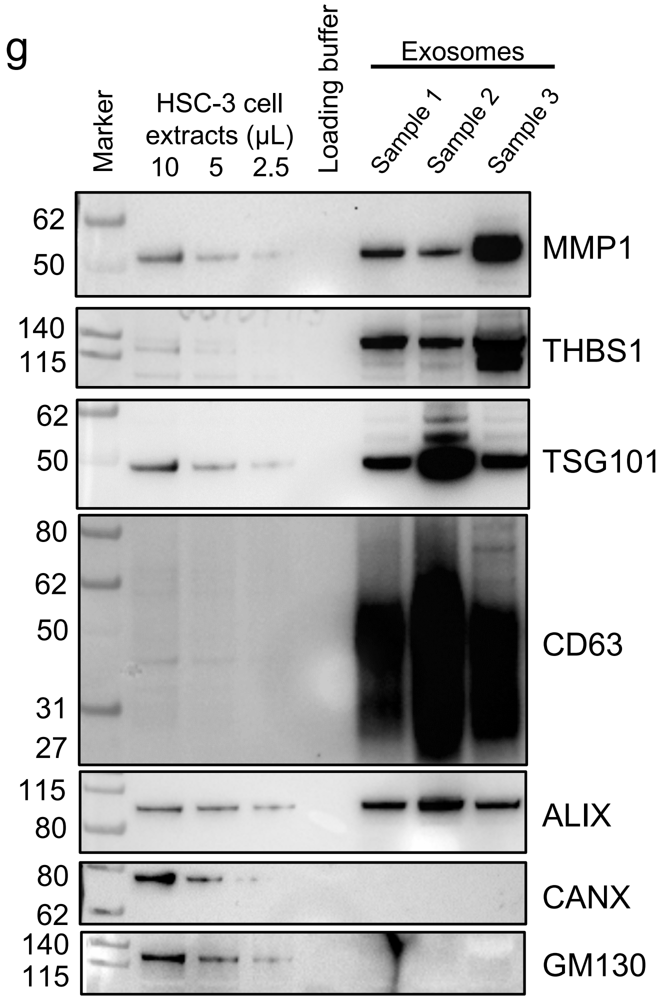


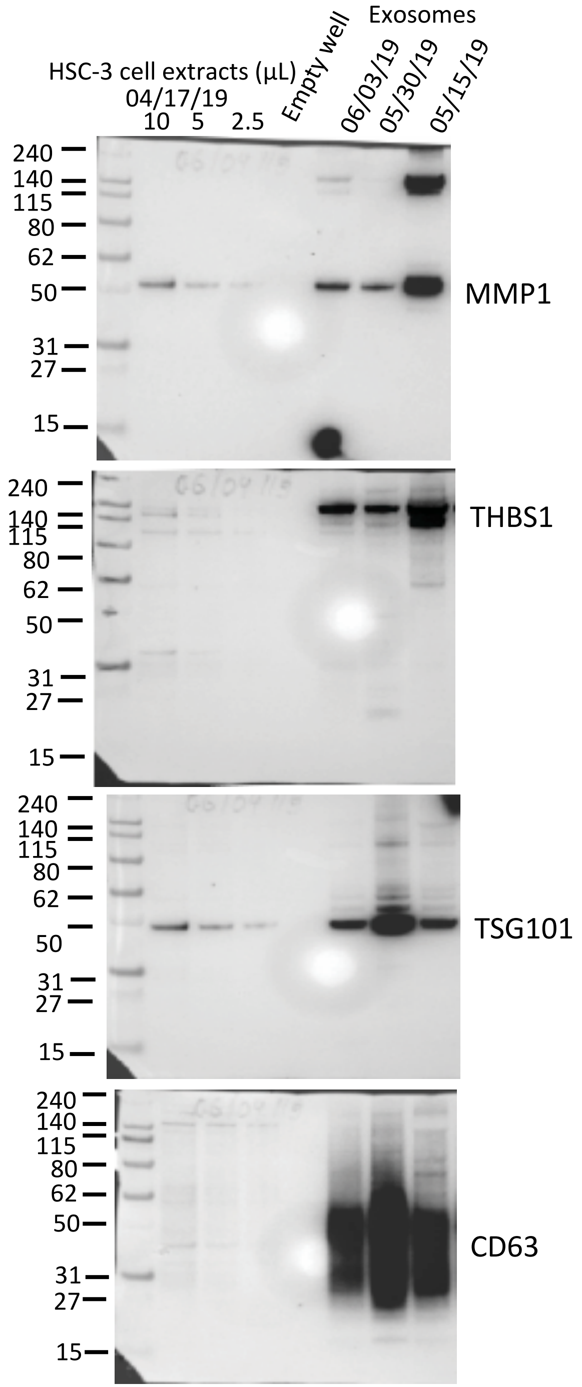


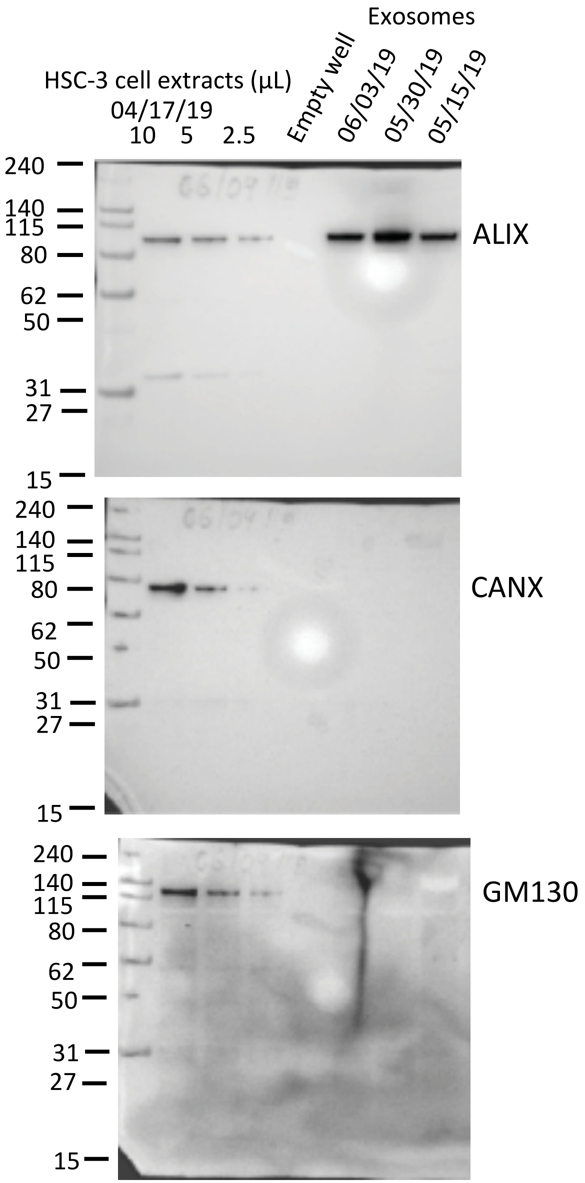

Figure 5

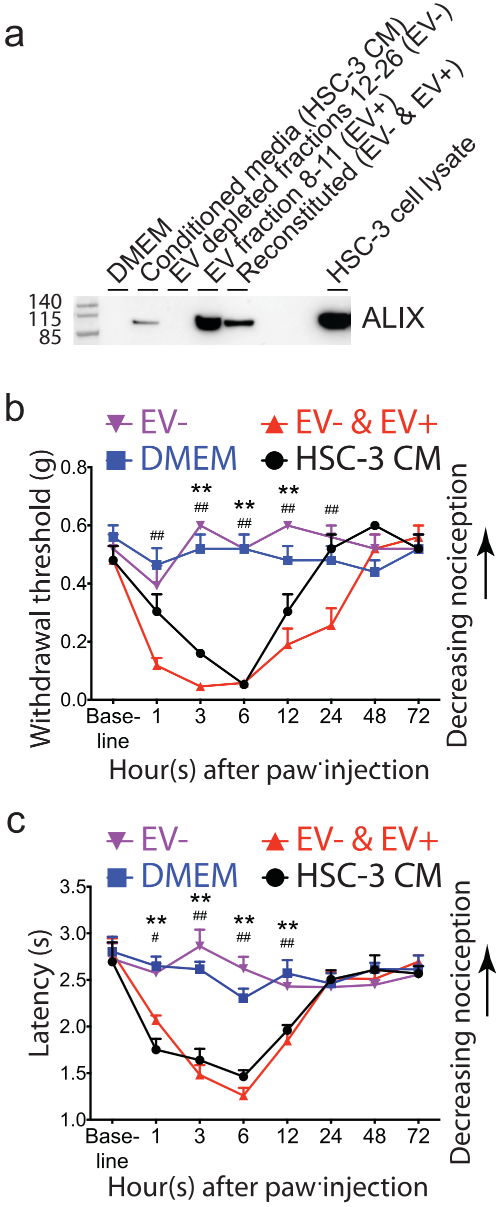


Western blot for Figure 5a

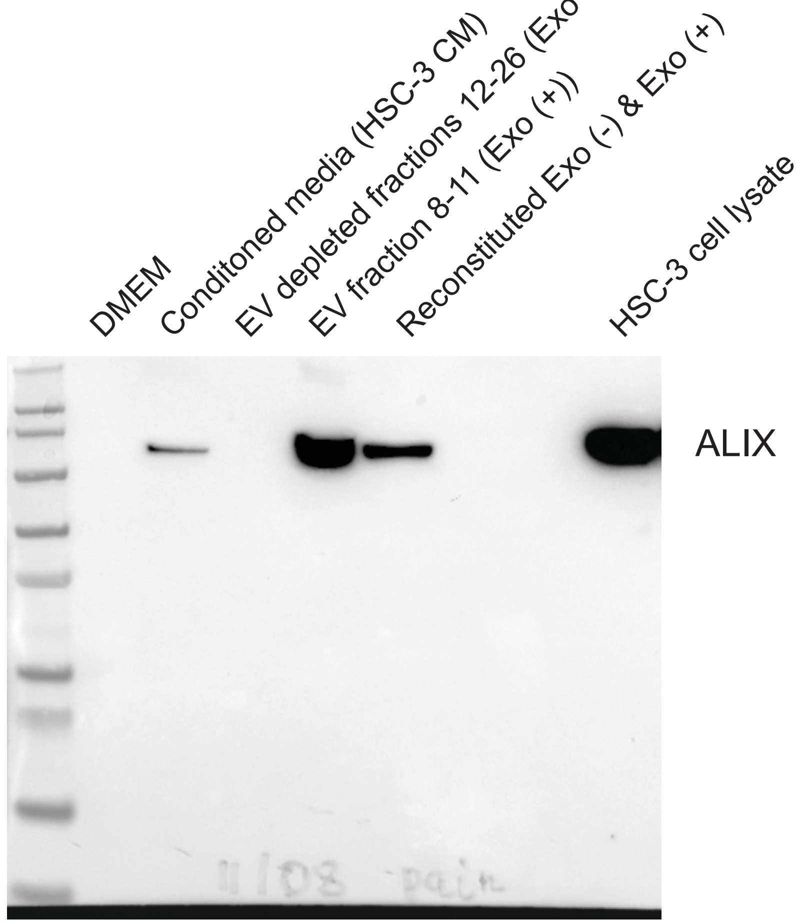

Figure S3 Exosomes from OSC-20 cells

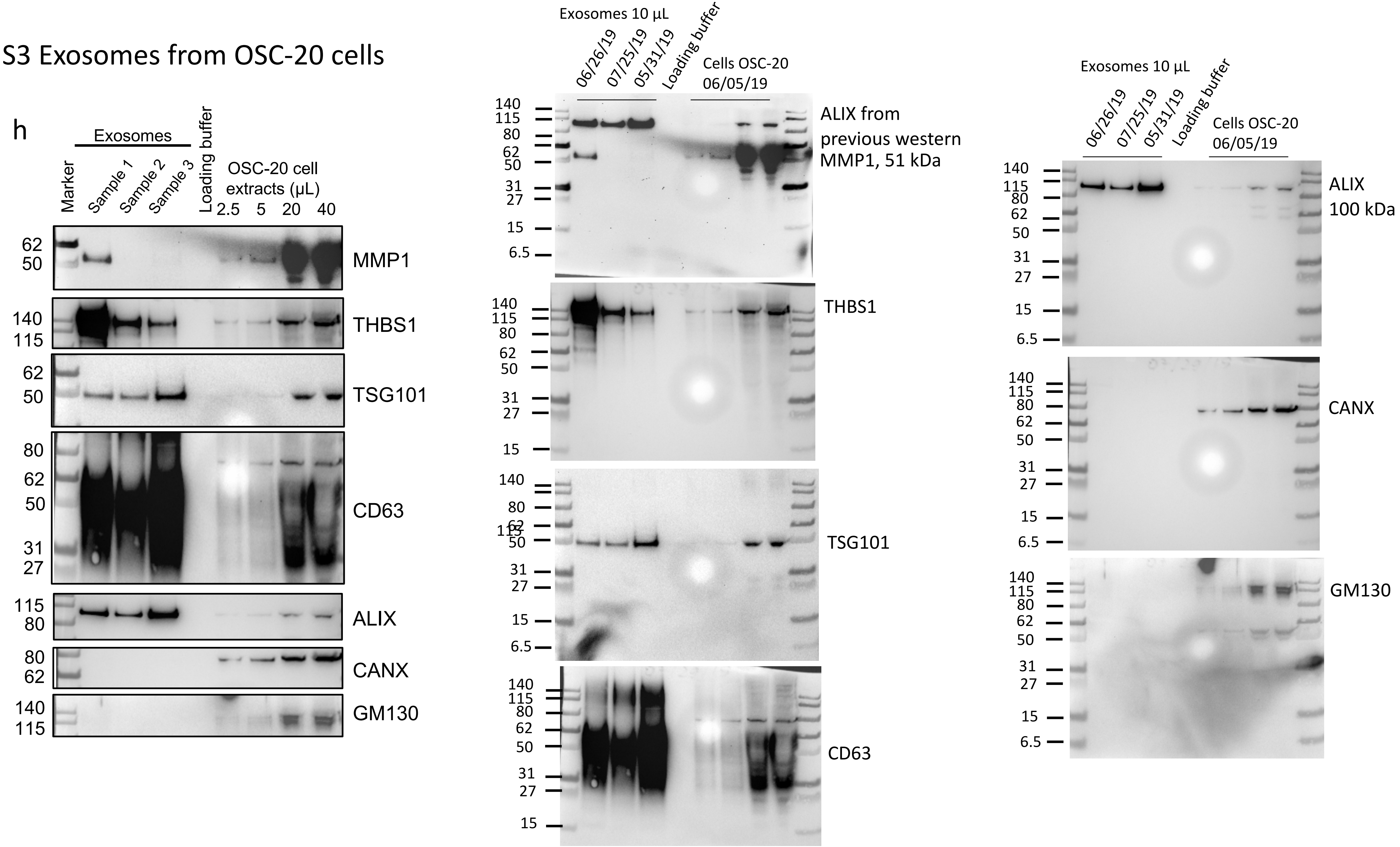

Supplementary Figure S4

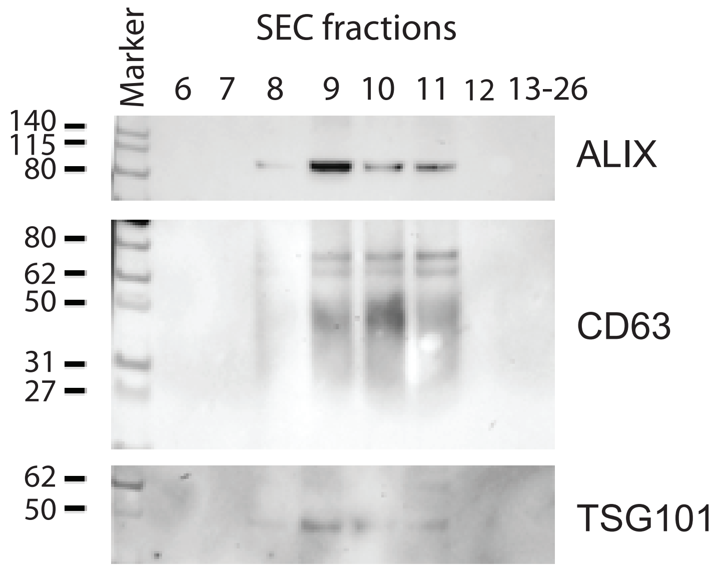


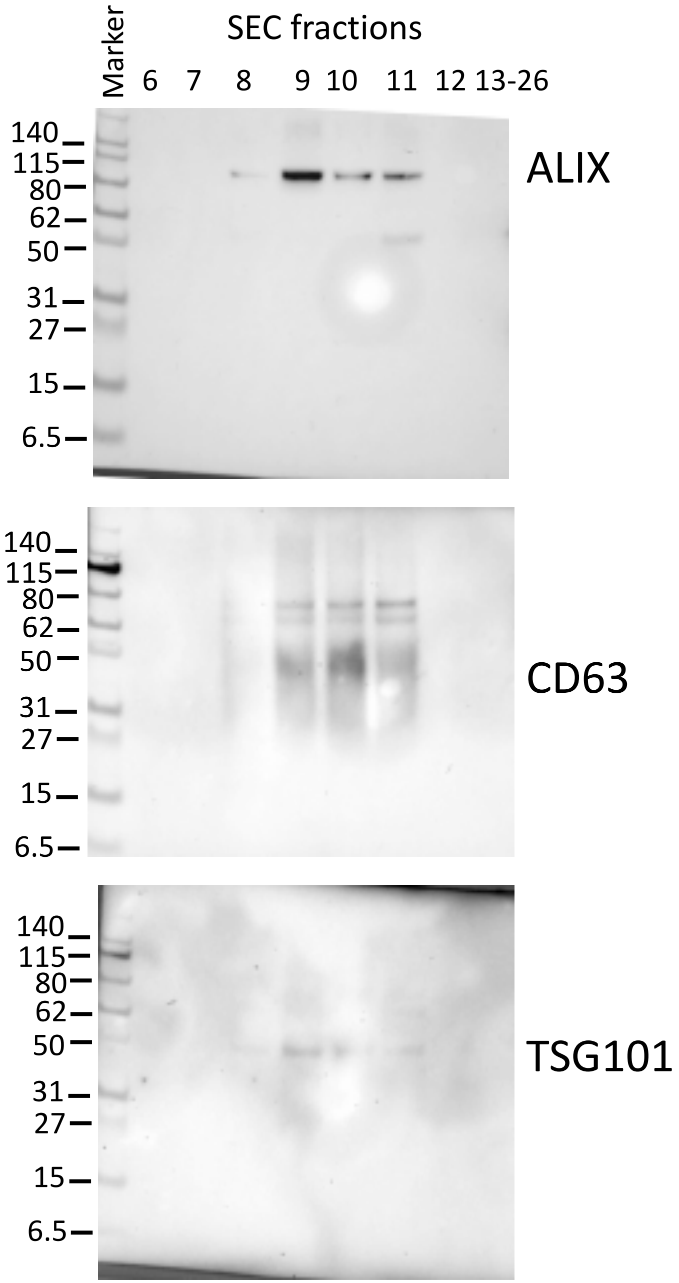

Supplement: Supplementary file 8 — Supplementary Figures [file 41598_2020_71298_MOESM8_ESM.pdf]
